# Association of Birth by Cesarean Delivery With Obesity and Type 2 Diabetes Among Adult Women

**DOI:** 10.1001/jamanetworkopen.2020.2605

**Published:** 2020-04-13

**Authors:** Jorge E. Chavarro, Nerea Martín-Calvo, Changzheng Yuan, Mariel Arvizu, Janet W. Rich-Edwards, Karin B. Michels, Qi Sun

**Affiliations:** 1 Department of Nutrition, Harvard T.H. Chan School of Public Health, Boston, Massachusetts; 2 Department of Epidemiology, Harvard T.H. Chan School of Public Health, Boston, Massachusetts; 3 Channing Division of Network Medicine, Department of Medicine, Brigham and Women’s Hospital and Harvard Medical School, Boston, Massachusetts; 4 Department of Preventive Medicine and Public Health, University of Navarra, Pamplona, Spain; 5 Physiopathology of Obesity and Nutrition, Carlos III Institute of Health, Madrid, Spain; 6 Instituto de Investigación Sanitaria de Navarra, Pamplona, Spain; 7 Connors Center for Women’s Health and Gender Biology, Brigham and Women’s Hospital and Harvard Medical School, Boston, Massachusetts; 8 Department of Epidemiology, Fielding School of Public Health, University of California, Los Angeles; 9 Institute for Prevention and Cancer Epidemiology, Faculty of Medicine and Medical Center, University of Freiburg, Freiburg im Breisgau, Germany

## Abstract

**Importance:**

Cesarean delivery is associated with an increased risk of childhood obesity in offspring. However, whether this increased risk also includes obesity-associated conditions remains unclear.

**Objective:**

To evaluate the association of birth by cesarean delivery with offspring’s risks of obesity and type 2 diabetes in adulthood.

**Design, Setting, and Participants:**

This prospective cohort study compared the incidence of obesity and type 2 diabetes between birth by cesarean delivery and vaginal delivery among 33 226 women participating in the Nurses’ Health Study II who were born between 1946 and 1964, with follow-up through the end of the 2013-2015 follow-up cycle. Participants’ mothers provided information on mode of delivery and pregnancy characteristics. Participants provided information every 2 years on weight and diagnosis of type 2 diabetes. Relative risks of obesity and type 2 diabetes were estimated using log-binomial and proportional hazards regression accounting for maternal body mass index and other confounding factors. Statistical analysis was performed from June 2017 to December 2019.

**Exposure:**

Birth by cesarean delivery compared with birth by vaginal delivery.

**Main Outcomes and Measures:**

Risk of obesity and incidence of type 2 diabetes.

**Results:**

At baseline, the participants’ mean (SD) age was 33.8 (4.6) years (range, 24.0-44.0 years). A total of 1089 of the 33 226 participants (3.3%) were born by cesarean delivery. After 1 913 978 person-years of follow-up, 12 156 (36.6%) women were obese and 2014 (6.1%) had received a diagnosis of type 2 diabetes. Women born by cesarean delivery were more likely to be classified as obese and to have received a diagnosis of type 2 diabetes during follow-up. The multivariable-adjusted relative risk of obesity among women born by cesarean vs vaginal delivery was 1.11 (95% CI, 1.03-1.19). The multivariable-adjusted hazard ratio for type 2 diabetes among women born by cesarean vs vaginal delivery was 1.46 (95% CI, 1.18-1.81); this association remained significant after additional adjustment for participant’s own body mass index (relative risk, 1.34 [95% CI, 1.08-1.67]). These associations persisted when analyses were restricted to women at low risk of cesarean delivery based on maternal characteristics.

**Conclusions and Relevance:**

This study suggests that women born by cesarean delivery may have a higher risk than women born by vaginal delivery of being obese and developing type 2 diabetes during adult life.

## Key Points

**Question:**

Is birth by cesarean delivery associated with obesity and type 2 diabetes in adulthood?

**Findings:**

In this cohort study of 33 226 women, those born by cesarean delivery were 11% more likely to be obese as adults and had a 46% higher risk of developing type 2 diabetes than women born by vaginal delivery.

**Meaning:**

This study found an association between being born by cesarean delivery and increased risks of obesity and type 2 diabetes in adulthood.

## Teaser

This cohort study evaluates the association of birth by cesarean delivery with offspring’s risks of obesity and type 2 diabetes in adulthood.

## Introduction

More than 1.2 million cesarean deliveries are performed yearly in the United States, making it the most common inpatient surgical procedure and accounting for nearly one-third of births nationwide.^1,2^ Starting at approximately 2.6% of all births in the 1930s (prior to the widespread availability of penicillin) and remaining stable around 5% between the 1950s^3^ and 1970s,^4^ the cesarean delivery rate in the United States rose to 24% in 1986,^4^ reaching a peak of 33% in 2009 and stabilizing around 30% thereafter,^1^ with primary cesarean delivery accounting for 50% of the increasing rate.^5^ When indicated, cesarean delivery is a lifesaving intervention to mother and fetus.^5,6,7,8^ Like all surgical procedures, however, cesarean deliveries are not without risks. Women without medical or obstetric risk factors for obstetric complications undergoing a planned cesarean delivery at term experience a 3-fold greater risk of major morbidity—including greater risks of cardiac arrest, hysterectomy, puerperal infection, and thromboembolism—relative to comparable women undergoing vaginal deliveries.^9^ For newborns, the most common immediate risk with cesarean delivery is a higher frequency of respiratory complications.^10,11^ Moreover, many cesarean deliveries performed in the United States do not have a clear indication,^12,13^ raising concerns that the excess maternal and newborn morbidity and mortality may be largely preventable. With these concerns in mind, leading professional organizations have advocated for the prevention of primary cesarean delivery as a strategy to reduce their overall frequency.^13,14^

Increasing evidence also suggests that being born by cesarean delivery may have long-term consequences on the health of offspring.^15,16,17,18^ One of the most consistent findings to date is that birth by cesarean delivery is associated with a higher risk of childhood obesity. Two meta-analyses summarizing data from 24 studies have reported an increased risk of obesity for individuals born by cesarean delivery (pooled odds ratio, 1.33 [95% CI, 1.19-1.48]^19^ and 1.22 [95% CI, 1.05-1.42]^20^), with little difference for risk of obesity during childhood vs adolescence and suggestive evidence that the elevated risk persists in adult life (odds ratio, 1.50 [95% CI, 1.02-2.20]).^19^ Subsequent studies, including studies based on long-term follow-up of large populations with stringent control for maternal body mass index (BMI) and other confounders, replicated these results.^21,22^ It is not clear whether the increased risk of obesity may also be accompanied by an increased risk of metabolic syndrome or type 2 diabetes during adult life. In 1 study, birth by cesarean delivery was associated with higher BMI, total cholesterol, low-density lipoprotein cholesterol, and leptin levels at age 20 years,^23^ but other studies have not identified associations between cesarean delivery and markers of metabolic risk.^24^ To further investigate the long-term association of birth by cesarean delivery with obesity and metabolic risk, we evaluated the association of cesarean delivery with obesity and incidence of type 2 diabetes in an ongoing prospective cohort study followed up for nearly 3 decades.

## Methods

### Study Population

The Nurses’ Health Study II (NHS-II) is an ongoing prospective cohort study established in 1989 when 116 671 female nurses aged 24 to 44 years enrolled in the study. Participants completed a self-administered questionnaire regarding lifestyle factors, anthropometric variables, and disease prevalence at recruitment. Updated information is collected through follow-up questionnaires every 2 years. In 2001, 39 904 mothers of NHS-II participants completed a questionnaire regarding their pregnancy with their NHS-II participant daughter and additional information regarding their daughter’s infancy, forming the Nurses’ Mothers Cohort Study.^25^ After excluding participants who did not provide information on height or weight, were not born of a singleton pregnancy, and whose mothers did not provide information on delivery mode, the final study sample included 33 226 NHS-II participants born between 1946 and 1964, with follow-up through the end of the 2013-2015 follow-up cycle. The study was approved by the Harvard School of Public Health and Brigham and Women’s Hospital Institutional Review Boards. In follow-up questionnaires, participants are informed in writing of the risks and benefits of participating in the study, and of their rights as participants; returning a completed questionnaire is considered evidence of informed consent. This study followed the Strengthening the Reporting of Observational Studies in Epidemiology (STROBE) reporting guideline.

### Exposure Assessment

Mode of delivery (cesarean vs vaginal delivery) was reported by the participants’ mothers in 2001. A validation study conducted among 154 women enrolled in the Collaborative Perinatal Project found perfect maternal recall of cesarean delivery at a mean of 32 years after delivery.^26^

### Ascertainment of Outcomes

At baseline, participants reported their height and weight, which are validly reported by adults,^27^ and updated this information every 2 years. Body mass index was calculated from these data as weight in kilograms divided by height in meters squared. We defined obesity (BMI ≥30) using the World Health Organization cutoffs.^28^

Participants reporting physician-diagnosed type 2 diabetes on follow-up questionnaires were mailed a supplemental questionnaire to confirm diagnoses. Cases of type 2 diabetes were confirmed based on the following American Diabetes Association criteria^29^: (1) 1 or more classic symptoms (excessive thirst, polyuria, weight loss, hunger, pruritus, or coma) plus elevated glucose levels (fasting plasma glucose concentration 126 mg/dL or more or random plasma glucose 200 mg/dL or more [to convert glucose to millimoles per liter, multiply by 0.0555]), or (2) no symptoms reported but 2 or more elevated plasma glucose concentrations on more than 1 occasion (fasting, 126 mg/dL or more; random, 200 mg/dL or more; or 2-hour oral glucose tolerance test, 200 mg/dL or more), or (3) treatment with insulin or an oral hypoglycemic agent. Before 1998, a fasting plasma glucose concentration of 140 mg/dL or more was used instead of 126 mg/dL or more for the diagnosis of diabetes according to the criteria of National Diabetes Data Group.^30^ In a validation study, a high consistency (98%) was observed comparing questionnaire-confirmed cases of type 2 diabetes against confirmation by medical record review.^31^ Participants were followed up from enrollment until the diagnosis of type 2 diabetes, death, or completion of the 2013-2015 follow-up cycle, whichever came first.

### Assessment of Covariates

Information on race/ethnicity, maternal educational level, maternal prepregnancy BMI, gestational weight gain, diagnosis of gestational diabetes, preeclampsia or gestational hypertension, gestational age, birth weight, and smoking during pregnancy was reported by participants’ mothers in 2001. A validation study showed that long-term maternal recall of many pregnancy-related events, including diagnosis of pregnancy complications (hypertensive disorders of pregnancy, gestational diabetes, placental abruption, and placenta previa), offspring birth weight, gestational age at delivery, smoking status, prepregnancy anthropometry, and gestational weight gain, were very accurately reported.^26^ Region of residence at birth was reported by NHS-II participants. Maternal age at delivery was calculated as the difference, in years, between participants’ date of birth and their mother’s date of birth. Missing indicators were used whenever data were missing for a covariate.

### Statistical Analysis

Statistical analysis was performed from June 2017 to December 2019. Maternal and offspring characteristics were presented according to delivery mode for which we used a Kruskal-Wallis test for continuous variables and a χ^2^ test for categorical variables when estimating differences between delivery modes. All *P* values were from 2-sided tests and results were deemed statistically significant at *P* < .05. To evaluate the association between cesarean delivery and offspring’s risk of obesity, we calculated relative risks (RRs) and 95% CIs using log-binomial regression models, or log-Poisson models when log-binomial models did not converge.^2^ To assess the association between cesarean delivery and offspring’s risk of type 2 diabetes, we calculated hazard ratios (HRs) using Cox proportional hazards regression models. We obtained crude and multivariable-adjusted estimates of these associations. The multivariable-adjusted models included terms for the following maternal variables: age at delivery (continuous, in years), race/ethnicity (white or other), educational level (≤high school or ≥college), prepregnancy BMI group (<18.5, 18.5-24.99, 25-29.99, or ≥30), gestational weight gain (continuous, in pounds), height (continuous, in inches), gestational diabetes (yes or no), preeclampsia (yes or no), pregnancy-induced hypertension (yes or no), year of birth (1946-1951, 1952-1961, or 1961-1963), gestational age at delivery (<37, 37-39, 40-42, or ≥43 weeks), birth weight group (<2.3, 2.3-3.1, 3.2-3.8, 3.9-4.4, ≥4.5 kg), smoking during pregnancy (no, first trimester, or second and third trimesters), and region of residence at birth (Northeast, Midwest, West, or South).

We conducted sensitivity analyses to address the possibility of residual confounding and evaluate the robustness of the findings. We fitted marginal structural models where the probability of cesarean delivery was assessed for each woman based on baseline characteristics and subsequently used to weight each observation using stabilized weights.^32,33^ In addition, we fitted models where we adjusted for maternal BMI with a linear and a quadratic term (instead of categories), restricted the obesity case definition to individuals who remained obese during follow-up, and allowed obesity status to vary over time. When assessing the association of cesarean delivery with the risk of type 2 diabetes, we additionally adjusted for offspring breastfeeding and fit log-binomial models for risk. To evaluate whether BMI explained any association between cesarean delivery and type 2 diabetes, the multivariable-adjusted model was additionally adjusted for offspring BMI during follow-up. We also conducted analyses restricted to participants in low-risk categories for cesarean delivery based on maternal characteristics (ie, maternal prepregnancy BMI <25, no gestational diabetes, no hypertensive disorders of pregnancy, no smoking during pregnancy, maternal age <30 years, gestational age at delivery between 37 and 42 weeks, and birth weight between 2.3 and 4.4 kg). All analyses were conducted using SAS software, version 9.2 (SAS Institute Inc).

## Results

At baseline, the participants’ mean (SD) age was 33.8 (4.6) years (range, 24.0-44.0 years). Of the 33 226 participants in the study, 1089 (3.3%) were born by cesarean delivery. Participants’ mothers who delivered by cesarean method had a higher mean (SD) prepregnancy BMI than those who delivered vaginally (21.7 [3.0] vs 21.2 [2.5]), were older (mean [SD] age at delivery, 28.2 [5.6] vs 26.2 [4.9] years), and were more likely to have preeclampsia (54 of 1089 [5.0%] vs 1018 of 32 137 [3.2%]), pregnancy-induced hypertension (55 of 1089 [5.1%] vs 1089 of 32 137 [3.4%]), preterm birth (64 [9.2%] vs 757 [4.1%]), and low birth weight (94 of 1089 [8.6%] vs 1903 of 32 137 [5.9%]) (Table 1). They were also less likely than those who delivered vaginally to smoke during pregnancy (249 of 1089 [22.9%] vs 8394 of 32 137 [26.1%]) and to breastfeed their participant daughters (350 of 1089 [32.1%] vs 14 820 of 32 137 [46.1%]). We documented 12 156 cases of obesity and 2014 new cases of type 2 diabetes during 1 913 978 person-years of follow-up. The cumulative risk of obesity through the end of follow-up was 36.5% (11 722 of 32 137) among women born by vaginal delivery and 39.9% (434 of 1089) among women born by cesarean delivery. The incidence of type 2 diabetes per 10 000 person-years was 10.4 among participants born by vaginal delivery and 14.1 among participants born by cesarean delivery.

**Table 1.** Maternal and Offspring Characteristics According to Mode of Delivery

Characteristic	All participants (N = 33 226)	Mode of delivery		*P* value^a^
		Vaginal (n = 32 137)	Cesarean (n = 1089)	
**Maternal characteristics**				
Age at delivery, mean (SD), y	26.3 (4.9)	26.2 (4.9)	28.2 (5.6)	<.001
Prepregnancy BMI, mean (SD)	21.2 (2.5)	21.2 (2.5)	21.7 (3.0)	<.001
Height, mean (SD), cm	163.3 (6.1)	163.6 (6.1)	160.8 (6.4)	.06
White race, No. (%)	32 321 (97.3)	31 265 (97.3)	1056 (97.0)	.53
Geographic region of birth, No. (%)				
Northeast	10 932 (32.9)	10 568 (32.9)	364 (33.4)	.32
Midwest	12 081 (36.4)	11 705 (36.4)	376 (34.5)	
West	3120 (9.4)	3009 (9.4)	121 (11.1)	
South	4061 (12.2)	3932 (12.2)	129 (11.9)	
Missing	3022 (9.1)	2923 (9.1)	99 (9.1)	
Gestational diabetes, No. (%)	140 (0.4)	133 (0.4)	7 (0.6)	.14
Preeclampsia, No. (%)	1072 (3.2)	1018 (3.2)	54 (5.0)	<.001
Gestational hypertension, No. (%)	1144 (3.4)	1089 (3.4)	55 (5.1)	<.001
Smoking during pregnancy, No. (%)				
No	24 583 (74.0)	23 743 (73.9)	840 (77.1)	.05
Yes, first trimester	1233 (3.7)	1197 (3.7)	36 (3.3)	
Yes, second and third trimesters	7410 (22.3)	7197 (22.4)	213 (19.6)	
Educational level, No. (%)				
≤High school degree	21 036 (63.3)	20 371 (63.4)	665 (61.1)	.10
Some college or college degree	12 071 (36.3)	11 654 (36.3)	417 (38.3)	
Missing	119 (0.4)	112 (0.4)	7 (0.6)	
Gestational weight gain, No. (%)				
<9.1 kg	10 920 (32.9)	10 526 (32.8)	394 (36.2)	.06
≥9.1 kg	19 699 (59.3)	19 088 (59.4)	611 (56.1)	
Missing	2607 (7.9)	2523 (7.9)	84 (7.7)	
**Offspring characteristics**				
Year of birth, No. (%)				
1946-1951	10 382 (31.3)	10 096 (31.4)	286 (26.3)	<.001
1952-1961	19 634 (59.1)	18 969 (59.0)	665 (61.1)	
1962-1964	3210 (9.7)	3072 (9.6)	138 (12.7)	
Gestational age at delivery, No. (%)^b^				
<37 wk	821 (4.3)	757 (4.1)	64 (9.2)	<.001
37-39 wk	8954 (46.8)	8498 (46.1)	456 (65.7)	
40-42 wk	7220 (37.7)	7111 (38.6)	109 (15.7)	
≥43 wk	2136 (11.2)	2071 (11.3)	65 (9.4)	
Birth weight group, No. %				
<2.3 kg	1997 (6.0)	1903 (5.9)	94 (8.6)	<.001
2.3-3.1 kg	10 731 (32.3)	10 313 (32.1)	418 (38.4)	
3.2-3.8 kg	18 657 (56.2)	18 135 (56.4)	522 (47.9)	
3.9-4.4 kg	1544 (4.7)	1495 (4.7)	49 (4.5)	
≥4.5 kg	297 (0.9)	291 (0.9)	6 (0.6)	
Breastfeeding duration, No. (%)				
Never	17 837 (53.7)	17 103 (53.2)	734 (67.4)	<.001
≤6 mo	11 419 (34.4)	11 159 (34.7)	269 (24.7)	
>6 mo	3751 (11.3)	3661 (11.4)	81 (7.4)	
Missing	219 (0.7)	214 (0.7)	5 (0.5)	

Abbreviation: BMI, body mass index (calculated as weight in kilograms divided by height in meters squared).

^a^ The 2-sample *t* test was used to test the difference of continuous variables and the χ^2^ test was used to test the difference of categorical variables.

^b^ For 19 131 participants.

Being born by cesarean delivery was associated with a higher risk of obesity (RR, 1.09 [95% CI, 1.01-1.18]) (Table 2). This association persisted in multivariable-adjusted analyses (adjusted RR, 1.11 [95% CI, 1.03-1.19]) and was similar across strata of age. Results were also similar when analyzing data using marginal structural models (RR, 1.11 [95% CI, 1.03-1.19]), when maternal prepregnancy BMI was modeled as a continuous variable (RR, 1.11 [95% CI, 1.03-1.19]), when case definition was restricted to women whose BMI remained ≥30 in all follow-up cycles after obesity was first documented (RR, 1.13 [95% CI, 1.05-1.22]), and when obesity status was allowed to change in each follow-up cycle (RR, 1.18 [95% CI, 1.08-1.29]).

**Table 2.** Association Between Mode of Delivery With Obesity in Offspring Among 33 226 Women

Mode of delivery	Obese participants, No./total No. (%)^a^	Offspring obesity	
		RR (95% CI)	*P* value
Overall	12 156/33 226 (36.6)	NA	NA
Vaginal	11 722/32 137 (36.5)	1 [Reference]	NA
Cesarean	434/1089 (39.9)		
Crude		1.09 (1.01-1.18)	.02
Adjusted^b^		1.11 (1.03-1.19)	.005
Additional analysis			
Marginal structural model estimate	434/1089 (39.9)	1.11 (1.03-1.19)	<.001
Maternal BMI as continuous variable^c^	434/1089 (39.9)	1.11 (1.03-1.19)	.006
Allowing obesity status to change over time	434/1089 (39.9)	1.18 (1.08-1.29)	<.001
Women who remained obese during follow-up (n = 31 867)	391/1046 (37.4)	1.13 (1.05-1.22)	.002

Abbreviations: BMI, body mass index (calculated as weight in kilograms divided by height in meters squared); NA, not applicable; RR, relative risk.

^a^ Body mass index of 30 or higher.

^b^ Adjusted models included terms for maternal age at delivery, race/ethnicity (white or other), maternal educational level (≥high school or ≥college), maternal prepregnancy BMI group (<18.5, 18.5-24.99, 25-29.99, or ≥30), gestational weight gain (<9.1 kg or ≥9.1 kg), maternal height, gestational diabetes (yes or no), preeclampsia (yes or no), pregnancy-induced hypertension (yes or no), year of birth (1946-1951, 1952-1961, or 1962-1964), gestational age at delivery (<37, 37-39, 40-42, or ≥43 weeks), birth weight group (<2.3, 2.3-3.1, 3.2-3.8, 3.9-4.4, or ≥4.5 kg), smoking during pregnancy (no, first trimester, second and third trimesters, or current), and region of residence at birth (Northeast, Midwest, West, or South).

^c^ Adjusted model modeling prepregnancy BMI as a continuous variable with a linear term and a quadratic term.

**Figure 1.**
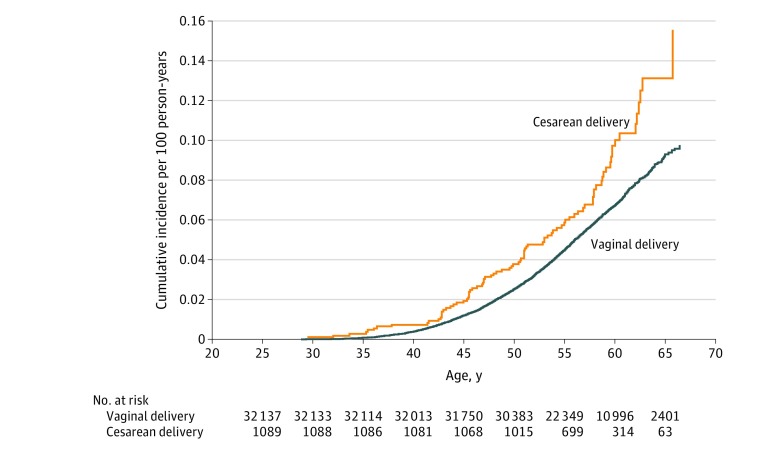
Cumulative Incidence of Type 2 Diabetes Among Women Born by Vaginal vs Cesarean Delivery

The incidence of type 2 diabetes was also higher among women born by cesarean delivery than among women born by vaginal delivery (Figure 1). The HR for type 2 diabetes in women born by cesarean vs vaginal delivery was 1.42 (95% CI, 1.14-1.76) (Table 3). This association persisted after multivariable adjustment (HR, 1.46 [95% CI, 1.18-1.81]), when analyzing data using marginal structural models (HR, 1.19 [95% CI, 0.96-1.50]), when maternal prepregnancy BMI was modeled as a continuous variable (HR, 1.47 [95% CI, 1.18-1.82]), and when risk of obesity was modeled using log-binomial models (HR, 1.49 [95% CI, 1.19-1.87]). Adjustment for breastfeeding did not change the association (HR, 1.45 [95% CI, 1.17-1.80]). Adjustment for updated offspring BMI status attenuated the association by 12% but the association remained statistically significant (HR, 1.34 [95% CI, 1.08-1.67]).

**Table 3.** Association Between Mode of Delivery With the Risk of Type 2 Diabetes

Characteristic	HR (95%CI)		*P* value
	Vaginal delivery	Cesarean delivery	
Cases of type 2 diabetes, No.	1927	87	NA
Person-time, Person-years	1 852 102	61 876	NA
Incidence rate of type 2 diabetes, per 10 000 person-years	10.4	14.1	NA
Crude	1 [Reference]	1.42 (1.14-1.76)	.002
Adjusted^a^	1 [Reference]	1.46 (1.18-1.81)	.001
Additional analyses			
Marginal structural model estimate	1 [Reference]	1.19 (0.96-1.50)	.12
Treating maternal BMI as continuous variable^b^	1 [Reference]	1.47 (1.18-1.82)	.001
Log-binomial model for risk ratio	1 [Reference]	1.49 (1.19-1.87)	<.001
Additional adjustment for offspring characteristics			
Breastfeeding	1 [Reference]	1.45 (1.17-1.80)	.001
Updated BMI	1 [Reference]	1.34 (1.08-1.67)	.008

Abbreviations: BMI, body mass index (calculated as weight in kilograms divided by height in meters squared); HR, hazard ratio; NA, not applicable.

^a^ Adjusted models included terms for maternal age at delivery, race/ethnicity (white or other), maternal educational level (≥high school or ≥college), maternal prepregnancy BMI group (<18.5, 18.5-24.99, 25-29.99, or ≥30), gestational weight gain (<9.1 kg or ≥9.1 kg), maternal height, gestational diabetes (yes or no), preeclampsia (yes or no), pregnancy-induced hypertension (yes or no), year of birth (1946-1951, 1952-1961, or 1962-1964), gestational age at delivery (<37, 37-39, 40-42, or ≥43 weeks), birth weight group (<2.3, 2.3-3.1, 3.2-3.8, 3.9-4.4, or ≥4.5 kg), smoking during pregnancy (no, first trimester, second and third trimesters, or current), and region of residence at birth (Northeast, Midwest, West, or South).

^b^ Adjusted model modeling prepregnancy BMI as a continuous variable with a linear term and a quadratic term.

**Figure 2.**
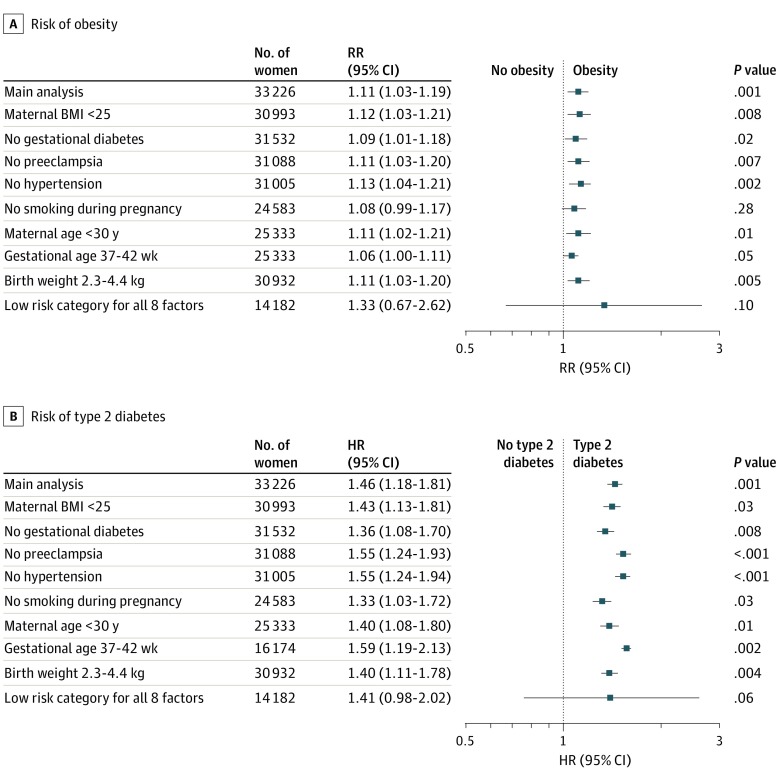
Associations of Birth by Cesarean Delivery With Risks of Offspring Obesity and Type 2 Diabetes Among Women in Low-Risk Categories for Cesarean Delivery Based on Maternal Characteristics A, Risk of obesity among offspring in adulthood. B, Risk of type 2 diabetes among offspring in adulthood. BMI indicates body mass index (calculated as weight in kilograms divided by height in meters squared); HR, hazard ratio; and RR, relative risk.

The associations of cesarean delivery with risks of obesity and type 2 diabetes were of comparable magnitude across each of the low-risk categories for cesarean delivery based on maternal characteristics, separately and when all were simultaneously considered (Figure 2). However, estimates were no longer statistically significant when analyses were restricted to women in all 8 low-risk groups.

## Discussion

In this prospective cohort of women aged of 24 to 66 years during the entire follow-up period, we found that being born by cesarean delivery was associated with an 11% higher risk of obesity and a 46% higher risk of type 2 diabetes. The association of cesarean delivery with type 2 diabetes persisted after adjustment for BMI during follow-up, suggesting that the association with type 2 diabetes is only partly mediated by elevated BMI. Findings were consistent across multiple strategies to account for confounding, suggesting that these associations are consistent with a true biological association of birth by cesarean delivery. Whether these findings are applicable to men or to individuals born today, when cesarean delivery rates are substantially higher, is uncertain.

Although the mechanisms for the development of obesity and type 2 diabetes in adulthood among individuals born by cesarean delivery remains unclear, growing evidence points to the hygiene theory and changes in the offspring’s gut microbiota.^34,35^ Gut microbiota can modulate host energy harvest from the diet and bacterial lipopolysaccharide-induced chronic inflammation. Thus, changes in gut microbiota can be associated with host adiposity and glucose metabolism.^36^ Mode of delivery is associated with the diversity in gut microbiota of the offspring.^37,38,39,40^ Vaginally delivered neonates are rapidly colonized by microbes from their mother’s birth canal and feces, while neonates delivered by cesarean delivery are colonized by environmental microbes.^36^ As a result, neonates born by cesarean delivery harbor a less diverse gut microbiota, particularly less *Bifidobacteria* and less *Bacteroides* spp, which have shown to be protective against obesity.^36,40^ Differences in gut microbiota composition by mode of delivery have been described in infants^40,41^ and children up to 7 years of age.^42^ Whether these differences are sustained long-term is unknown. Differences in DNA methylation patterns between children born by cesarean delivery and those born by vaginal delivery have also been proposed as a biological explanation underlying long-term health outcomes of cesarean delivery, but data are scarce.^43,44,45,46^ Higher global DNA methylation has been reported in infants born by cesarean delivery,^43,44^ including a study in which a genome-wide analysis identified 343 loci that were nominally (*P* < .01) differentially methylated between infants born by cesarean delivery and those born by vaginal delivery.^44^ Other studies, however, have found no difference in DNA methylation between children born by vaginal delivery or cesarean delivery.^45,46^

We found that, compared with those born by vaginal delivery, offspring born by cesarean delivery had an 11% higher risk of obesity and a 46% higher risk of type 2 diabetes in adulthood. Our finding of an association with obesity in adulthood is in agreement with the results of 2 meta-analyses, which reported an increased risk in offspring obesity of 22%^20^ and 50%,^19^ and with more recent prospective cohorts that included young adults and reported associations of similar magnitude to that reported here.^21^ However, to our knowledge, an association of birth by cesarean delivery with risk of type 2 diabetes has not been previously described. Two prospective cohort studies^24,47^ previously reported that cesarean delivery was not associated with metabolic risk factors in offspring after 20 to 23 years of follow-up. On the other hand, a recent prospective cohort study^23^ found that, compared with those born by vaginal delivery, young adults born by cesarean delivery showed a more adverse cardiometabolic risk profile. More important, participants in these 3 studies were all in their early 20s and the prevalence of type 2 diabetes may not have been sufficiently high to identify differences in prevalence by mode of delivery. In fact, the incidence data from our study (Figure 1) suggest that differences in risk of type 2 diabetes may not become evident until the fourth decade of life. Although, to our knowledge, our study has the largest sample size and longest duration of follow-up of all studies addressing the association of cesarean delivery with type 2 diabetes to date, these findings should be interpreted with caution given that it is the first time that an association with type 2 diabetes is reported and very few studies have examined the association with obesity-related metabolic abnormalities. Further research is needed to replicate the association with type 2 diabetes and address the biological mechanisms underlying the association of cesarean delivery with the increased risk of offspring obesity and type 2 diabetes in adulthood.

### Limitations and Strengths

Our study has some limitations. The most important limitation is the lack of data on indications for cesarean delivery. Historical data^3^ suggest that the most common indications of cesarean delivery during the time period in which participants in this study were born were cephalopelvic disproportion, previous cesarean delivery, and placenta previa, jointly accounting for approximately 80% of cesarean deliveries.^3^ These data further suggest that no less than 30% and as much as half of all cesarean deliveries during this period were planned and presumably performed before the onset of labor. Although we cannot determine whether these figures apply to our study participants, they do highlight the importance of our analyses restricted to low-risk groups, as these analyses may have eliminated many cesarean deliveries performed owing to cephalopelvic disproportion. Moreover, other common indications of cesarean delivery during this period, namely labor arrest and breech presentation, are not known risk factors for offspring obesity and are therefore unlikely to be important confounders of the association of cesarean delivery with offspring obesity or type 2 diabetes. The individuals in this study are nurses participating in a long-term health study; while this facilitated long-term follow-up and the prospective collection of high-quality detailed data, it may hamper the generalizability of the findings to the general population. The fact that maternal report of mode of birth and other pregnancy-related information was retrospective and thus subject to recall bias could be reasonably identified as a major potential limitation. However, cesarean delivery rates in this cohort are comparable to cesarean delivery rates in the general population at the time participants were born^4^ and, as previously mentioned, past studies have shown perfect recall of cesarean delivery and highly accurate reporting of other pregnancy events and important potential confounders (including smoking status during pregnancy, prepregnancy weight, and gestational weight gain) decades later.^26^ An additional limitation is the underrepresentation of minorities in our cohort. However, there are no a priori reasons to believe these associations would differ across race or ethnicity. We also acknowledge the possibility of residual confounding owing to the lack of information about potentially relevant covariates such as the use of antibiotics during pregnancy or intrapartum and birth order. We also lacked information on offspring gut microbiota, DNA methylation patterns, or other potential biological mediators to further explore the underlying mechanisms.

It is also important to consider the generalizability of the findings to current practice in light of the differences in cesarean delivery rates at the time of the study and today. As this is an observational study, further research is needed before causality can be assumed. Nevertheless, the current study has multiple strengths and was able to address the most salient limitations of previous studies. The prospective study design, large sample size, and long-term follow-up allowed us to examine the associations of cesarean delivery and the risk of obesity and type 2 diabetes of the offspring in adulthood, and to provide precise estimates of these associations. The availability of key prepregnancy and pregnancy information allowed for multiple sensitivity analyses aimed at addressing residual confounding.

## Conclusions

We observed associations of cesarean delivery with increased risks of obesity and type 2 diabetes of the offspring in adulthood. Most important, the association remained significant in most of the analyses restricted to participants in low-risk categories for cesarean delivery based on maternal characteristics. Greater evidence from large, prospective studies with high-quality data on prepregnancy, pregnancy, and delivery information (particularly information regarding the timing of cesarean delivery relative to the onset of labor or rupture of membranes), as well as data from studies with sibling pairs discordant in delivery mode, is needed to address if these findings are generalizable and to investigate whether offspring born by cesarean delivery are at a higher risk of developing other adverse metabolic and cardiovascular outcomes.
